# Supplementary material for: Social determinants of healthy aging: An investigation using the all of us cohort
Source: PLoS One. 2026 Mar 6;21(3):e0342292. doi: 10.1371/journal.pone.0342292 (PMC12965612; doi:10.1371/journal.pone.0342292)
Supplement: S1 Table — (DOCX) [file pone.0342292.s001.docx]

S1. Performance metrics for the three algorithms

| **S1. Performance metrics of the testing datasets** | | | | | | |
| --- | --- | --- | --- | --- | --- | --- |
| **Primary Cohort** | **Under-sampling** | | | **Over-sampling** | | |
|  | **LR** | **XGB** | **MLP** | **LR** | **XGB** | **MLP** |
| AUROC | 0.786 | 0.796 | 0.790 | 0.786 | 0.795 | 0.794 |
| F1 score | 0.704 | 0.716 | 0.716 | 0.702 | 0.717 | 0.717 |
| Recall | 0.766 | 0.806 | 0.822 | 0.765 | 0.803 | 0.814 |
| Precision | 0.650 | 0.645 | 0.634 | 0.649 | 0.648 | 0.640 |
| Accuracy | 0.715 | 0.718 | 0.712 | 0.713 | 0.720 | 0.716 |
|  | | | | | | |
| **Secondary Cohort** | **Under-sampling** | | | **Over-sampling** | | |
|  | **LR** | **XGB** | **MLP** | **LR** | **XGB** | **MLP** |
| AUROC | 0.850 | 0.796 | 0.854 | 0.857 | 0.795 | 0.886 |
| F1 score | 0.411 | 0.716 | 0.408 | 0.418 | 0.717 | 0.442 |
| Recall | 0.807 | 0.806 | 0.826 | 0.802 | 0.803 | 0.869 |
| Precision | 0.276 | 0.645 | 0.271 | 0.283 | 0.648 | 0.297 |
| Accuracy | 0.754 | 0.718 | 0.745 | 0.762 | 0.720 | 0.767 |
