# Supplementary material for: Social determinants of healthy aging: An investigation using the all of us cohort
Source: PLoS One. 2026 Mar 6;21(3):e0342292. doi: 10.1371/journal.pone.0342292 (PMC12965612; doi:10.1371/journal.pone.0342292)
Supplement: S2 Table — (DOCX) [file pone.0342292.s002.docx]

S2. Bootstrapped performance on the test dataset over 50 iterations

|  | AUROC  (95% CI) | AUPRC  (95% CI) | F1 score  (95% CI) | Recall  (95% CI) | Precision  (95% CI) | Accuracy  (95% CI) |
| --- | --- | --- | --- | --- | --- | --- |
| **Primary Cohort** | | | | | | |
| XGBoost with RUS | 0.792  (0.788-0.796) | 0.707  (0.700-0.713) | 0.697  (0.692-0.701) | 0.742  (0.733-0.749) | 0.658  (0.654-0.662) | 0.716  (0.711-0.719) |
| XGBoost with ROS | 0.793  (0.788-0.796) | 0.708  (0.700-0.714) | 0.697  (0.692-0.701) | 0.739  (0.732-0.748) | 0.659  (0.655-0.663) | 0.716  (0.712-0.720) |
| LR with RUS | 0.785  (0.781-0.789) | 0.697  (0.691-0.702) | 0.703  (0.700-0.707) | 0.772  (0.766-0.778) | 0.646  (0.642-0.652) | 0.713  (0.709-0.717) |
| LR with ROS | 0.785  (0.781-0.789) | 0.698  (0.692-0.703) | 0.679  (0.675-0.685) | 0.696  (0.691-0.703) | 0.663  (0.657-0.668) | 0.710  (0.705-0.714) |
| MLP with RUS | 0.791  (0.787-0.795) | 0.708  (0.702-0.714) | 0.704  (0.693-0.715) | 0.767  (0.729-0.798) | 0.651  (0.641-0.662) | 0.716  (0.711-0.720) |
| MLP with ROS | 0.793  (0.789-0.796) | 0.710  (0.705-0.716) | 0.699  (0.689-0.709) | 0.745  (0.716-0.775) | 0.658  (0.650-0.666) | 0.717  (0.712-0.721) |
| **Secondary Cohort** | | | | | | |
| XGBoost with RUS | 0.793  (0.789-0.795) | 0.707  (0.702-0.710) | 0.697  (0.693-0.700) | 0.741  (0.733-0.746) | 0.658  (0.655-0.660) | 0.716  (0.714-0.718) |
| XGBoost with ROS | 0.793  (0.788-0.796) | 0.708  (0.700-0.714) | 0.697  (0.692-0.701) | 0.739  (0.732-0.748) | 0.659  (0.655-0.663) | 0.716  (0.712-0.720) |
| LR with RUS | 0.857  (0.854-0.862) | 0.389  (0.379-0.398) | 0.238  (0.235-0.240) | 0.154  (0.151-0.156) | 0.518  (0.510-0.524) | 0.895  (0.894-0.895) |
| LR with ROS | 0.857  (0.854-0.862) | 0.389  (0.379-0.398) | 0.238  (0.235-0.240) | 0.154  (0.151-0.156) | 0.518  (0.510-0.524) | 0.895  (0.894-0.895) |
| MLP with RUS | 0.855  (0.848-0.863) | 0.387  (0.363-0.406) | 0.099  (0.050-0.143) | 0.054  (0.026-0.082) | 0.599  (0.493-0.731) | 0.895  (0.893-0.897) |
| MLP with ROS | 0.860  (0.854-0.868) | 0.397  (0.373-0.417) | 0.138  (0.116-0.161) | 0.079  (0.065-0.095) | 0.574  (0.514-0.648) | 0.896  (0.894-0.898) |

*AUROC: Area under the Receiver Operating Characteristic Curve, AUPRC: Area Under the Precision-Recall Curve, XGBoost: extreme gradient boosting, LR: logistic regression, MLP: multilayer perception, RUS: random undersampling, ROS: random oversampling
