# Supplementary material for: Social determinants of healthy aging: An investigation using the all of us cohort
Source: PLoS One. 2026 Mar 6;21(3):e0342292. doi: 10.1371/journal.pone.0342292 (PMC12965612; doi:10.1371/journal.pone.0342292)
Supplement: S3 Table — (DOCX) [file pone.0342292.s003.docx]

S3. Selection of model hyperparameters using Bayesian optimization with 5-fold cross-validation

| **Hyperparameter** | **Tuning range for Bayesian optimization** | **Optimal value chosen** |
| --- | --- | --- |
| **Primary cohort** | | |
| Column sample by tree | Real (0.5, 1, ‘log-uniform’) | 0.5 |
| Learning rate | Real (0.1, 0.5, ‘log-uniform’) | 0.1 |
| Gamma | Real (1e-1, 1, ‘log-uniform’) | 0.14924415627138107 |
| Maximum tree depth | Integer (3, 10) | 9 |
| Minimum child weight | Integer (1, 20) | 8 |
| Number of estimators | Integer (10, 500) | 419 |
| Alpha | Real (1e-5, 100, ‘log-uniform’) | 7.80142448939489 |
| Lambda | Real (1e-5, 100, ‘log-uniform’) | 100.0 |
| Subsample | Real (0.1, 1, ‘log-uniform’) | 1.0 |
| **Secondary cohort** | | |
| Column sample by tree | Real (0.5, 1, ‘log-uniform’) | 0.5236057037849238 |
| Learning rate | Real (0.1, 0.5, ‘log-uniform’) | 0.1 |
| Gamma | Real (1e-1, 1, ‘log-uniform’) | 0.5904806515456753 |
| Maximum tree depth | Integer (3, 10) | 10 |
| Minimum child weight | Integer (1, 20) | 10 |
| Number of estimators | Integer (10, 500) | 429 |
| Alpha | Real (1e-5, 100, ‘log-uniform’) | 0.06936620412496958 |
| Lambda | Real (1e-5, 100, ‘log-uniform’) | 49.25072571620478 |
| Subsample | Real (1e-5, 100, ‘log-uniform’) | 1.0 |
