# Supplementary material for: Social determinants of healthy aging: An investigation using the all of us cohort
Source: PLoS One. 2026 Mar 6;21(3):e0342292. doi: 10.1371/journal.pone.0342292 (PMC12965612; doi:10.1371/journal.pone.0342292)
Supplement: S4 Table — (DOCX) [file pone.0342292.s004.docx]

S4. Confusion matrix for the primary cohort

S4a. Confusion matrix for Black and White

|  |  | Predicted | |
| --- | --- | --- | --- |
|  | Black total: 14612 | Healthy aging  2109 | Non-healthy aging  12503 |
| Actual | Healthy aging  3597 | 1223 | 2374 |
|  | Non-healthy aging  11015 | 886 | 10129 |

|  |  | Predicted | |
| --- | --- | --- | --- |
|  | White total: 67457 | Healthy aging  40985 | Non-healthy aging  26472 |
| Actual | Healthy aging  33916 | 27472 | 6444 |
|  | Non-healthy aging  33541 | 13513 | 20028 |

S4b. Confusion matrix for male and female

|  |  | Predicted | |
| --- | --- | --- | --- |
|  | Male total: 41977 | Healthy aging  23582 | Non-healthy aging  18395 |
| Actual | Healthy aging  20493 | 15790 | 4703 |
|  | Non-healthy aging  21484 | 7792 | 13692 |

|  |  | Predicted | |
| --- | --- | --- | --- |
|  | Female total: 55294 | Healthy aging  24152 | Non-healthy aging  31142 |
| Actual | Healthy aging  22455 | 15941 | 6514 |
|  | Non-healthy aging  32839 | 8211 | 24628 |
